# Short-term outcomes and clinical efficacy of stereotactic body radiation therapy (SBRT) in treatment of adrenal gland metastases from lung cancer

**DOI:** 10.1186/s13014-018-1152-5

**Published:** 2018-10-22

**Authors:** Xianzhi Zhao, Xiaofei Zhu, Jianfeng Fei, Haipeng Ren, Yangsen Cao, Xiaoping Ju, Zhiyong Yuan, Huojun Zhang

**Affiliations:** 1 Department of Radiation Oncology, Shanghai Changhai Hospital, the Second Military Medical University, Shanghai, 200433 China; 2 Department of Radiology, Shanghai Changhai Hospital, the Second Military Medical University, Shanghai, 200433 China; 3 Department of Oncology, Weifang People’s Hospital, Weifang, 261041 China; 4 Department of Radiation Oncology and CyberKnife Center, Key Laboratory of Cancer Prevention and Therapy, Tianjin’s Clinical Research Center for Cancer, Tianjin Medical University Cancer Institute and Hospital, National Clinical Research Center for Cancer, Tianjin, 300060 China

**Keywords:** Adrenal gland metastases, Stereotactic body radiotherapy (SBRT), Lung cancer, Local control, Response

## Abstract

**Background:**

To assess the efficacy and safety of stereotactic body radiation therapy (SBRT) in the management of adrenal gland metastases (AGMs) from lung cancer. Moreover, it is the first two-institutional experience and the largest-to-date study to report the safety and efficacy of SBRT for inoperable AGM from lung cancer.

**Methods:**

In this retrospective study, 30 patients (27 males, 3 females) with 32 AGMs were treated by SBRT from October 2006 to June 2016. Of these, 11 patients were treated with the intent of controlling all known metastatic sites and 19 for palliation of bulky AGMs. Follow-up was performed every 3 months for evaluations of efficacy and safety. Factors predictive of overall survival (OS) and local control (LC) were identified with univariate and then multivariate analysis.

**Results:**

Median follow-up time was 10.7 months (2.9–96.4 months). The complete response (CR), partial response (PR), stable disease (SD) and progressive disease (PD) rates were 23.3%, 33.3%, 33.3% and 16.7% respectively. The 6-month, 1, and 2-year LC rates were 96.9%, 96.9%, and 72.7% respectively. Additionally, the 6-month, 1, and 2-year OS rates were 85.6%, 58.1%, and 54.0% respectively while 6-month, 1, and 2-year progression free survival (PFS) rates were 39.5%, 24.6%, and 8.2%, respectively. All the patients with cancer-induced pain (8 with abdominal pain and 6 with lumbar back pain) had significant alleviations after SBRT. The treatment was well tolerated with only 1 patient reporting grade-3 diarrhoea. No predictors of OS and LC were found after multivariate analysis, while it was demonstrated that biologic equivalent dose (BED_10_, α/β = 10) ≥85.5Gy (*P* = 0.007) and gross tumor volume < 30 ml (*P* = 0.003) correlated with LC only after univariate analysis.

**Conclusion:**

SBRT is a safe and effective treatment modality in the management of AGMs from lung cancer with high LC rates and acceptable toxicity.

## Background

The adrenal gland is one of the most frequently involved sites of metastases in lung cancer. Adrenal gland metastases (AGMs) from lung cancer are usually characterized by insidious onset and patients only occasionally have back or abdominal pain due to a large or rapidly growing tumor. Rarely, in the case of bilateral adrenal involvement, patients may develop adrenal insufficiency, which may result in attenuation of quality of life (QOL) and often worse survival [1]. A new radiographically evidence of tumor in the adrenal gland contributed to early diagnosis of AGM during follow-up [2].

An obvious increase in tumor burden may prompt systemic aggressive treatment. However, for those with AGMs who may have already received first-line chemotherapy and have disease progressions thereafter, alternative regimens might not provide survival benefits, as well as probably contribute to high incidences of toxicity. Furthermore, though the adverse effects of targeted therapy for patients with EGFR mutation are mild, drug resistance remains the biggest problem. Surgery is considered as a curative option for some isolated metastases, but the adrenalectomy sometimes may result in adrenal insufficiency and perioperative complications [3]. Percutaneous image-guided radiofrequency ablation (RFA) is a safe and well-tolerated modality for unresectable primary or metastatic adrenal gland tumor, which greatly contributes to short-term local control, especially for the tumors with diameters less than 5 cm [4]. Nevertheless, RFA is still micro-invasive option. Hence, a non-invasive, safe and effective alternative is required.

Recently, due to the advantages of stereotactic body radiation therapy (SBRT), including precise delivery and abrupt dose fall-off outside targets and high local dose conformation, it has been commonly used in selected patients with and without metastatic lesions, with high local control (LC) rates and acceptable toxicity, especially when surgery is declined or contraindicated [5–7]. High doses could be precisely delivered to an extracranial target within the body, either as a single dose or a small limited number of radiation fractions [8]. It can be performed either with a traditional linear accelerator or a robotic arm (CyberKnife®). CyberKnife was developed in the 1990s at Stanford (Accuray Inc., Sunnyvale, CA, USA) [9], which represented an innovations of traditional stereotactic surgery. Given the real-time tracking, beam angles could be simultaneously corrected intrafractionally via pre-identified patient’s breathing patterns [10]. Despite the previous employment of SBRT in the lung cancer, pancreatic cancer, prostate cancer and liver cancer [11–14], few studies investigated the management of AGMs from lung cancer with SBRT. The aim of this retrospective study was to assess the efficacy and safety of SBRT for AGMs from lung cancer.

## Methods

### Patient characteristics

SBRT was delivered for 30 patients with AGMs from lung cancer (total 32 lesions) from October 2006 to June 2016 at Tianjin Medical University Cancer Institute and Hospital, and Changhai Hospital of the Second Military Medical University. All patients were examined by an oncologist before the study. Patients with AGMs diagnosed by biopsy or at least imaging examinations. Besides, those with a Karnofsky performance score ≥ 70, a life expectancy of > 3 months and who declined or were not amenable to surgical resections due to comorbidities were included in the study. Informed consent was obtained from all patients prior to the enrolment and the study was conducted according to the Declaration of Helsinki. The review and use of medical records of patients for analysis were approved by the ethical committee.

### Treatments

Of the 30 patients, 11 were treated with the intent of controlling all known metastatic lesions, and 19 underwent SBRT for palliation of bulky adrenal metastases. SBRT was delivered by CyberKnife (Accuray Corporation, Sunnyvale, CA, USA). All patients were immobilized in supine position with arms by their sides using thermoplastic body mask. Computed tomography (CT) scan was performed with a slice thickness of 1.5 mm, with the scan range of at least 10 cm below and above the tumor. According to the adrenal gland motions during radiotherapy reported by Wang et al. [15], for patients with X-sight spine tracking, the gross tumor volume (GTV) was defines as a radiographically lesion in the adrenal gland. Planning target volume (PTV) was delineated with a 3–5 mm margin expansion in the lateral direction and anteroposterior direction, a 5–7 mm margin expansion in the cephalo-caudal direction from GTV. When Synchrony™ Respiratory Tracking System was used, PTV was determined by a 3 mm margin expansion in the lateral direction and anteroposterior direction, a 3–5 mm margin expansion in the cephalo-caudal direction. Synchrony™ Respiratory Tracking System was performed in 9 patients with 11 lesions, while X-sight spine tracking was employed in 21 patients with 21 lesions. The treatment parameters were presented in Table 1. The dose-volume constraints for organs at risk were referred to the American Association of Physicists in Medicine guidelines in TG-101 [16], which was as follows: renal hilum/vascular trunk, V_23_ < 2/3 volume; renal cortex (right and left), V_17.5_ < 200 mL; duodenum, D_5mL_ < 18 Gy, D_10mL_ < 12.5 Gy and D_max_ < 32 Gy; liver V_21_ < 700 mL; spinal cord, D_0.35mL_ < 23 Gy, D_1.2mL_ < 14.5 Gy and D_max_ < 30 Gy; medulla spinal cord (5-6 mm above and below level treated per Ryu), V_23_ < 10% of subvolume and D_max_ < 30 Gy; stomach, D_10mL_ < 18 Gy and D_max_ < 32 Gy; duodenum, D_5mL_ < 18 Gy, D_10mL_ < 12.5 Gy and D_max_ < 32 Gy.

**Table 1:** Patient demography and clinical presentation

Characteristics	Values		
Age (years)	63(range 42–85)		
Gender (male/female)	27/3 (90%/10.0%)		
Karnofsky performance score ≥ 70	30 (100%)		
Pathology of primary tumor		Lesions per patient	
▪ Squamous cell carcinoma	7(23.3%)	▪ 1	28 (93.3%)
▪ Adenocarcinoma	12 (40.0%)	▪ > 1	2 (6.7%)
▪ Small cell lung cancer	5 (16.7%)	Systemic therapy after SBRT	
▪ Adenosquamous carcinoma	3 (10.0%)		
▪ Unknown	3 (10.0%)	▪ Yes	18 (60%)
Location of AGMs		▪ None	12 (40%)
▪ Left	16 (53.3%)	Metastases in other sites	
▪ Right	12 (40%)	▪ Yes	15 (50%)
▪ Left and right	2 (6.7%)	▪ No	15 (50%)
Symptoms		SBRT for other sites	
▪ Presented	14 (46.7%)	▪ Yes	9 (30%)
▪ None	16 (53.3%)	▪ No	21 (70%)

### Response evaluation and follow-up

Patients were re-evaluated every 3 months after radiotherapy with contrast-enhanced CT scans, PET-CT scans or contrast-enhanced MRI. Adverse events, amelioration of symptoms and sequential treatment were recorded. Acute and late toxicity was scored according to the Common Terminology Criteria for Adverse Events (CTCAE) version 4.0 [17]. Tumor response was defined as complete response (CR), partial response (PR) and stable disease (SD), which was determined using the Response Evaluation Criteria in Solid Tumors (RECIST), version 1.1 [18]. Therefore, the local control (LC) rate was the ratio of the number of lesions with the effective response from the completion of SBRT to a determined time point to the total number of lesions. OS was defined as the time from the date of the receipt of SBRT to death from any cause or the last follow-up for included patients. PFS was defined as the time from the date of the receipt of SBRT to the confirmation of disease progressions at any sites or death by any cause.

### Statistical analysis

LC, OS and PFS curves were calculated by the Kaplan-Meier method. Potential factors associated with LC, OS and PFS were identified with univariate log-rank comparisons and then multivariate proportional hazards regression model. Statistical analyses were performed using SPSS 18.0 (IBM Corporation, Armonk, NY, USA). Two-sided *P* values < 0.05 were considered statistically significant.

## Results

### Patient characteristics

Patient characteristics were shown in Table 1. A total of 30 patients with a median age of 63 years (range: 42–85), including 27 males and 3 females, were treated with CyberKnife® (Accuray Incorporated, Sunnyvale, CA, USA),. Of all patients, 16 and 12 lesions were in the left and right adrenal gland while 2 patients had bilateral AGMs without adrenal insufficiency due to quite small lesions. The patlatehological types of lung cancer included squamous cell carcinoma (*n* = 7, 23.3%), adenocarcinoma (*n* = 12, 40.0%), small cell lung cancer (*n* = 5, 16.7%), adenosquamous carcinoma (*n* = 3, 10.0%), and unknown type (n = 3, 10.0%). Among all the patients, 15 (50.0%) had other site metastases in addition to AGMs. Eighteen patients (60.0%) had received chemotherapy after SBRT, and 9 (30.0%) had more than one lesions treated. Furthermore, 16 patients (53.3%) were symptom-free, while 14 (46.7%) suffered from abdominal pain or lumbar back pain.

**Fig. 1:**
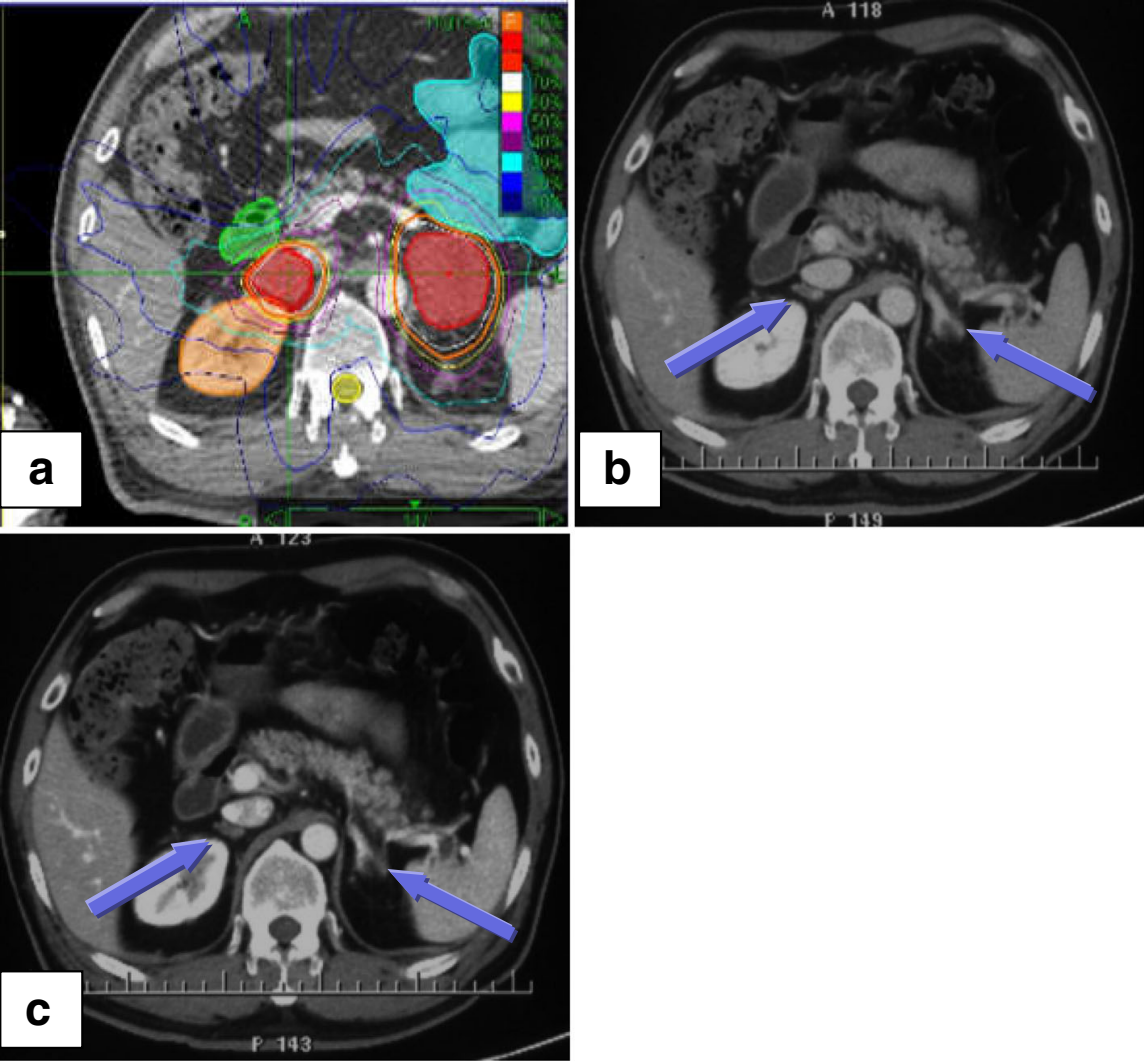
An illustrative case of successful SBRT for 51-year-old man with two AGMs from NSCLC. **a** CT scan before SBRT and 45 Gy in 5 fractions was prescribed for each AGMs. Red shadow areas represented bilateral GTV. **b** Enhanced CT scan one month after SBRT. **c** Enhanced CT scan three months after SBRT.GTV: gross tumor volume; AGMs: adrenal gland metastases; SBRT: stereotactic body radiation therapy; CT: computed tomography. Note: The arrows indicate AGMs after SBRT

The median dose was 44.4 Gy (range 32–50 Gy), which was delivered in 5 fractions (3–8 fractions), with a corresponding biologic effective dose (α/β = 10, BED_10_) of 85.5 Gy (range: 44.8–112.5 Gy). The median prescription dose for control and palliation intent was 45Gy (range: 42-50Gy) and 42Gy (range: 32-50Gy), respectively. The median GTV was 23.9 ml (range: 2.5–115.3 ml). Treatment planning parameters were demonstrated in Table 2. Additionally, an illustrative case was shown in Fig. 1.

**Table 2:** Treatment parameters used for SBRT

	All lesions	Lesions with local control	Lesions without local control
GTV (ml)	23.9 (2.5–115.3)	17.8 (2.5–75)	60.7 (26.4–115.3)
Maximum dose (Gy)	58 (43.2–70.4)	57.7 (43.2–70.4)	58.6 (44.9–66.2)
Total prescribed dose (Gy)	44.4 (32–50)	45 (32–50)	41.6 (35–45)
Number of fractions	5 (3–8)	5 (3–8)	5 (3–8)
Dose per fraction (Gy)	9 (4–15)	9 (4–15)	7 (5.2–15)
BED_10_ (Gy)	85.5 (44.8–112.5)	85.5 (44.8–112.5)	67.2 (59.5–112.5)
Prescription isodose line (%)	73 (66–82)	74 (66–82)	71 (66–79)

All data were shown as median values (range)

### Efficacy outcomes

**Fig. 2:**
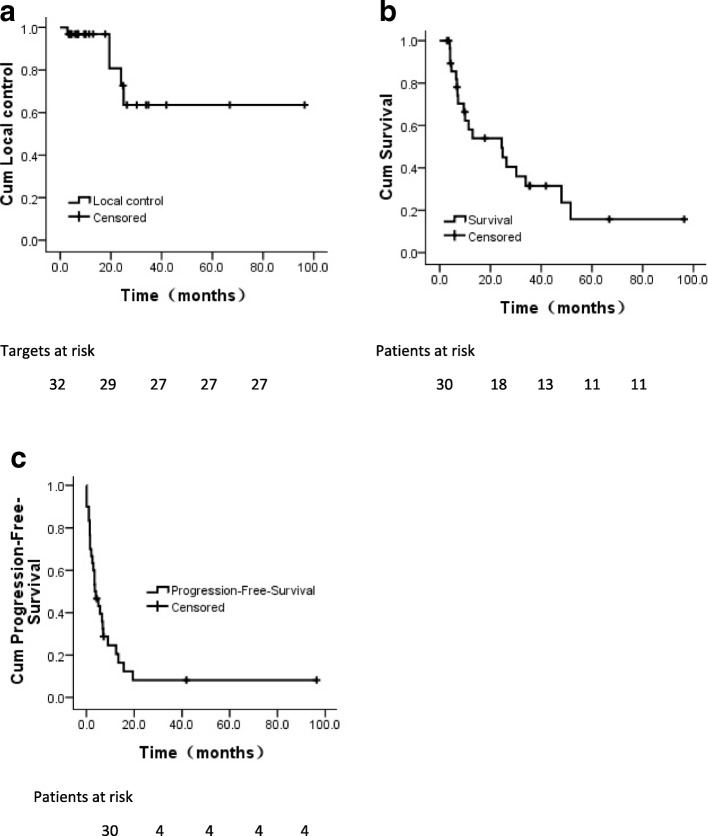
Actuarial survival analysis of patients. **a** Overall local control. **b** Overall survival in general. **c** Overall progression-free-survival. Cum, cumulative

All patients were followed up until death or June 2018. By the last follow-up, 19 patients (63.3%) died while 11 were alive. One patient died of heart disease, whereas 18 patients died of distant metastasis. Hence, local failure and radiation-induced toxicity did not contribute to the death. The median follow-up was 10.7 months (2.9–96.4 months), and median OS and PFS was 24.4 months (95% CI: 4.4–44.4 months) and 3.5 months (95% CI: 1.3–5.7), respectively. The 6-month, 1-year, and 2-year LC rates were 96.9%, 96.9%, and 72.7%, respectively (Fig. 2a). Furthermore, the 6-month, 1-year, and 2-year OS rates were 85.6%, 58.1%, and 54.0%, respectively (Fig. 2b), And the 6-month, 1-year, and 2-year PFS rates were 39.5%, 24.6%, and 8.2%, respectively (Fig. 2c). Tumor response of each lesion of the adrenal gland was evaluated. Therefore, based on the RECIST criteria, the CR, PR and SD rates were 21.9% (*n* = 7), 31.2% (*n* = 10), and 31.2% (*n* = 10) respectively, while 5 (15.7%) foci showed disease progression (PD).

Nineteen of thirty patients (21/32 targets) were treated for palliation purpose. The 6-month, 1, and 2-year LC rates of these targets were 95.2%, 95.2%, and 47.6% respectively, whereas the 6-month, 1, and 2-year OS rates of these patients were 76.0%, 54.9%, and 54.9% respectively. Elven of thirty patients (11/32 targets) were treated for radical purpose. The 6-month, 1, and 2-year LC rates of these targets were all 100%, whereas the 6-month, 1, and 2-year OS rates of these patients were 100%, 63.6%, and 54.5% respectively.

In the univariate analysis, patients with GTV < 30 ml had a high LC rate than those with GTV ≥ 30 ml (*P* = 0.003, Table 3). Moreover, BED_10_ ≥ 85.5Gy was predictive of better LC (*P* = 0.007, Table 3). However, no factors were found to be correlated with LC after the multivariate analysis.

**Table 3:** Univariate analysis for LC rate

	1-year LC rate (%)	2-year LC rate (%)	*P* Value
BED (Gy)			
<85.5	92.3	23.1	0.007
≥ 85.5	100	100	
Prescription dose (Gy)			
<42	100	25.0	0.072
≥ 42	95.5	95.5	
GTV (ml)			
<30	100	100	0.003
≥ 30	92.9	23.2	
Systemic therapy after SBRT			
Yes	100	70.0	0.913
No	92.9	92.9	
Tracking method			
X-sight spine	95.2	81.6	0.869
synchrony respiratory motion	100	60.0	

Similarly, no predictors were associated with OS after univariate and multivariate analysis (Table 4).

**Table 4:** Univariate analysis for OS rate

	1-year OS rate (%)	2-year OS rate (%)	*P* Value
Concurrence of metastasis in other sites			
Yes	59.4	59.4	0.740
No	57.1	50.0	
Systemic therapy after SBRT			
Yes	71.4	71.4	0.119
No	41.0	30.8	
Local control			
Yes	50.4	45.3	0.320
No	100	100	
Metastasis in other sites after SBRT			
Yes	56.7	52.0	0.164
No	66.7	66.7	
Age (years)			
<60	56.2	46.9	0.674
≥ 60	58.7	58.7	

Among the patients who had tumor-induced symptoms prior to the treatment (including 8 with abdominal pain and 6 with lumbar back pain), all of them (100.0%) had alleviations of symptoms after SBRT.

### Treatment toxicity

The treatment was well-tolerated. No grade 4 or higher acute toxicity was observed and only 1 patient had grade 3 diarrhea. Fatigue, poor appetite, nausea, thrombocytopenia, leucopenia, vomiting and pain were the most common side effects during treatment. There were no grade 3 or higher late gastrointestinal toxicities. Furthermore, even grade 1 or 2 late gastrointestinal toxicity was not recorded during follow-up.. All the adverse effects were temporary, reversible and ameliorated by medication, which did not prevent patients from completing the treatment (Table 5). Additionally, three patients and one patient received tyrosine-kinase inhibitors and anti-vascular endothelial growth factor agent. and only mild toxicities, including skin rash or diarrhea, were found and alleviated by supportive care.

**Table 5:** Adverse effects reported/observed

AE	Grade 1–2	Grade3 or more	Total *N* (%)
Nausea	10 (33.3%)	0 (−)	10 (33.3%)
Vomiting	2 (6.7%)	0 (−)	2 (6.7%)
Poor appetite	11 (36.7%)	0 (−)	11 (36.7%)
Diarrhea	2 (6.7%)	1 (3.3%)	3 (10.0%)
Hepatic injury	1 (3.3%)	0 (−)	1 (3.3%)
Renal injury	2 (6.7%)	0 (−)	2 (6.7%)
Leucopenia	5 (16.7%)	0 (−)	5 (16.7%)
Thrombocytopenia	6 (20.0%)	0 (−)	6 (20.0%)
Fatigue	14 (46.7%)	0 (−)	14 (46.7%)
Myalgia	2 (6.7%)	0 (−)	2 (6.7%)
Abdominal pain	11 (36.7%)	0 (−)	11 (36.7%)

## Discussion

The study investigated the efficacy of SBRT for AGMs from lung cancer. Overall, SBRT may provide high LC rates, survival benefits with the median OS of 24.4 months and symptom relief without serious toxicities. Additionally, no grade 4 or above acute toxicities were reported. Therefore, it may be implied that SBRT was a promising modality in the management of AGMs from lung cancer, especially for a palliative purpose to relieve various symptoms caused by the metastases.

Surgery was considered as a standard curative-intent strategy for isolated adrenal metastasis from non-small cell lung cancer (NSCLC). In the analysis of 13 retrospective studies, the median OS of patients with NSCLC and isolated adrenal metastasis treated with segmentectomy, lobectomy or pneumonectomy, and adrenalectomy was 18 months, and the 1-, 2- and 5-year survival rates were 66.5, 40.5 and 28.2%, respectively [19]. Another efficacious local-regional treatment was image-guided RFA. A retrospective study evaluated 35 patients with 41 AGMs with the mean size of 3.3 cm from various types of primary tumors. At the last follow-up, 27 patients (77%) achieved local control. The 1-, 3-, and 5-year OS rates were 75%, 34%, and 30% respectively, with a median survival time of 26.0 months [20]. Nevertheless, in our study, 15 patients had metastases in other sites in addition to AGMs. Moreover, there were 8 AGMs with the diameter more than 5 cm, 14 AGMs with the volume more than 30 ml. Hence, those patients may not be amenable to surgical resections and RFA. Due to quick dose fall-off and fewer low dose areas outside targets compared with conventional radiotherapy, SBRT may be beneficial for them, especially when they had failed in adjuvant chemotherapy, molecular targeted drug therapy, and conventional radiotherapy.

Recently, SBRT has been an alternative for patients with AGMs from different types of tumor [21–24]. In a Florence study [21], 48 patients, most of whom had AGMs from lung cancer, received SBRT. The median follow-up was 16.2 months. Both of the actuarial 1-year and 2-year LC rate were 90%, and the1-year and 2-year OS rates were 39.7% and 14.5%, respectively. The actuarial 1-year disease control rate was 9%. In another study, 30 patients with AGMs underwent SBRT. The 1-year OS, LC, and distant control rate was 44%, 55%, and 13%, respectively. No patients developed grade 2 or greater toxicity [22]. However, few studies focused on AGMs exclusively from lung cancer. In a relevant report, SBRT was performed in 18 patients with AGMs from NSCLC. The median follow-up was 12 months. Ten out of thirteen patients (77%) with an isolated lesion in the adrenal gland achieved local control with a median OS and PFS of 21 and 4.2 months, respectively [25]. Besides, another study reported the 1-year and 2-year local control rates of 15 patients after SBRT were 60% and 46.6%, respectively. The median OS was 17.3 months. One- and two-year disease free survival rate was 60% and 46.6%, respectively, while the median disease-free survival was 10.5 months [26]. Similar outcomes were also found in Franzese et al. with the median OS of 28.5 months and 1-year and 2-year LC rates of 65.5% and 40.7%, respectively [27]. Nevertheless, the median OS was inferior than those in the above studies when patients received SBRT or conventional radiotherapy [28], which might be attributable to lower BED_10_ compared with those of SBRT. The findings in current study were consistent with previous studies. The 0.5, 1, and 2-year LC rates were 96.9%, 96.9% and 72.7% respectively. The 0.5, 1, and 2-year OS rates were 85.6%, 58.1%, and 54.0% respectively, and similarly the corresponding PFS rates were 39.5%, 24.6%, and 8.2% respectively. Moreover, it was the first multicenter study to report the safety and efficacy of SBRT for inoperable AGMs from lung cancer.

A dose-response relationship has been observed in several studies about SBRT for AGMs. Previous studies demonstrated that BED_10_ < 60Gy was predictive of lower 1-year LC rates [22, 29, 30] while other reports identified that BED_10_ > 85Gy correlated with better LC [21, 31, 32]. In our study, patients with BED_10_ ≥ 85.5Gy had higher 1- and 2-year LC rates compared with those with BED_10_ < 85.5Gy (*P* = 0.007).

In our study, no late gastrointestinal or renal toxicities were found, which was consistent with the previous studies [25–28]. Only two patients developed multiple gastric or duodenal ulcer [25]. The low incidence of late toxicity may be attributable to the smaller tumor volume compared with that in Holy, Celik and Scorsetti et al. [25, 26, 28], though the radiation doses in our study were higher than those prescribed in all those studies.

In Zishan’ s study, they evaluated and examined the influence of tumor size on outcomes for medically inoperable early-stage non-small cell lung cancer after SBRT. As a result, tumor size was not associated with local failure but regional failure and distant failure [33]. However, in Doré’s study, they evaluated local control after postoperative hypofractionated stereotactic radiosurgery in patients with brain metastasis. On univariate analysis, initial tumour volume less than 9.5 cm^3^ (*P* = 0.05) and PTV less than 12 cm^3^ (*P* = 0.005) were associated with improved LC [34]. In our study, tumor size was an important predictor of local control. We observed patients who had smaller AGMs (GTV < 30 ml) had a better LC rate (*P* = 0.003) than those who had bigger ones (GTV ≥ 30 ml). Notably, it must be noted that high LC rates cannot be achieved with the compromise of protections of organs at risk. Therefore, high radiation doses may not be prescribed in the case of large tumors due to dose constraints of surrounding normal tissues, while smaller lesions may receive higher doses. Hence, a prospective trial is needed to confirm these preliminary observations.

Typically, abdominal pain and lumbar back pain were the two most common symptoms in patients with AGMs. We found that all of the patients had symptomatic relief after SBRT. A previous study reported that all 3 patients with AGMs rated their score as 0 on a 10-point scale, which were 4–5 before SBRT [22]. The results were consistent with our study. What is interesting and worth investigating is that since SBRT is effective in symptoms ameliorations, it might be indicated that SBRT could be employed soon after AGMs are found to prevent potential tumor-induced symptoms.

There were some limitations of this study. It was retrospective, and with a limited number of patients or targets. In addition, the treatment schedules were heterogeneous: there was a wide range of doses prescribed with various fractionations. Furthermore, a longer follow-up is warranted to examine the impact of LC on regional or distant disease control and survival.

## Conclusion

SBRT is an effective modality with fast symptom relief, and acceptable toxicity for lung cancer patients with AGMs. Patients with BED_10_ ≥ 85.5 Gy and GTV < 30 ml had a better LC rate. Because most patients died of or were at high risk of distant metastasis, local treatment combined with systemic treatment may contribute to a better outcome. Moreover, the efficacy and safety of SBRT for patients with AGMs from lung cancer needed to be validated in the prospective studies.

## Abbreviations

AGM: Adrenal gland metastase
BED: Biologic equivalent dose
CR: Complete response
CT: Computed tomography
CTCAE: Common terminology criteria for adverse events
DVH: Dose-volume histogram
GTV: Gross tumor volume
LC: Local control
NSCLC: Non-small cell lung cancer
OS: Overall survival
PD: Progressive disease
PFS: Progression free survival
PR: Partial response
PTV: Planning target volume
QOL: Quality of life
RECIST: Response evaluation criteria in solid tumors
RFA: Radiofrequency ablation
SBRT: Stereotactic body radiation therapy
SD: Stable disease
